# Multiphase optimization implementation strategy for tobacco cessation interventions in primary care clinics: A cluster-randomized type 3 hybrid effectiveness-implementation trial

**DOI:** 10.18332/tid/217143

**Published:** 2026-03-24

**Authors:** Jiangyun Chen, Jiao Yang, Siyuan Liu, Wenjun He, Xueying Chen, Yiyuan Cai, Guangyu Tong, Haozheng Zhou, Huanyuan Luo, Lingzi Luo, Dong Xu

**Affiliations:** 1 Acacia Lab for Implementation Science and Center for World Health Organization Studies, School of Health Management, Southern Medical University, Guangzhou, China; 2 Department of Global Health and Population, School of Public Health, Harvard University, Boston, United States; 3 School of Public Health, Capital Medical University, Beijing, China; 4 School of Public Health, Southern Medical University, Guangzhou, China; 5 Dermatology Hospital, Southern Medical University, Guangzhou, China; 6 School of Medicine and Health Management, Tongji Medical College, Huazhong University of Science and Technology, Wuhan, China; 7 Acacia Lab for Primary Healthcare, Department of Epidemiology and Health Statistics, School of Public Health, Guizhou Medical University, Guiyang, China; 8 Department of Internal Medicine, Yale School of Medicine, New Haven, United States; 9 Department of Biostatistics, Yale School of Public Health, New Haven, United States; 10 Shenzhen Hospital of Southern Medical University, Shenzhen, China; 11 School of Global Public Health, New York University, New York, United States

**Keywords:** brief verbal intervention for smoking cessation (BISC), primary healthcare, clinicians, multiphase optimization strategy (MOST), factorial trial

## Abstract

Brief verbal intervention for smoking cessation (BISC) is an evidence-based practice proven to be effective and cost-efficient, yet its implementation in primary healthcare (PHC) clinics in China is currently inadequate. Developing and evaluating multifaceted implementation strategies are crucial to translating such evidence-based practices into routine clinical care. This study protocol outlines a rigorous, multi-phase approach to identifying and testing the most effective strategies for BISC implementation. This is a two-phase study utilizing the Multiphase Optimization Strategy (MOST). The first phase, which has been completed, involved using a scoping review, stakeholder consultations, and a Best-Worst Scaling (BWS) online survey to systematically identify and define four key implementation strategies. The second phase is a cluster-randomized 2×2×2 factorial trial conducted in primary healthcare institutions in Guangdong province, China, from January 2026 to December 2026. Eligible participants are clinicians providing primary healthcare services. Clusters will be randomly assigned, and outcome assessors and data analysts will be blinded to allocation. Data collection includes unannounced standardized patient (USP) visits, questionnaires, interviews, and telephone follow-ups, with outcomes assessed six months after the endline. The study's outcomes are categorized into a primary aim (implementation outcomes) and a secondary aim (effectiveness outcomes). This protocol outlines the utilization of the MOST framework to customize the optimized combination of implementation techniques for BISC within the local context. By using a factorial design, the study could unpack the black box of multi-component interventions and identify which specific components are most effective and efficient. This approach is expected to provide a valuable methodological paradigm for advancing implementation science in low- and middle-income countries.

**CLINICAL TRIAL REGISTRATION:**

The study is registered on the official website of Chinese Clinical Trials

**IDENTIFIER:**

ChiCTR2600115994

**ABBREVIATIONS:**

BISC: brief verbal intervention for smoking cessation, PHC: primary healthcare, CFIR: Consolidated Framework for Implementation Research, BWS: best-worst scaling, MOST: Multiphase Optimization Strategy, USP: unannounced standardized patient, PSU: primary sampling unit, SSU: secondary sampling unit, PPS: probability proportional to size, DSMB: Data and Safety Monitoring Board, IOF: Implementation Outcomes Framework, PSAT: Program Sustainability Assessment Tool, PRESS: Provider Report of Sustainment Scale, NPT: Normalization Process Theory, EBP: evidence-based practice

## INTRODUCTION

Tobacco use poses a significant public health threat in China, the world’s largest tobacco producer and consumer^1^. With over 1 million annual tobacco-related deaths, projected to reach 2 million by 2030, important action is needed^2^. The Healthy China 2030 initiative aims to reduce adult smoking rates from 27.7% in 2015 to 20% by 2030^3^, necessitating the implementation of evidence-based smoking cessation practices.

Brief verbal intervention for smoking cessation (BISC), a 1-3 minute verbal advice delivered by health professionals during routine consultations, is a highly effective^4^ and a cost-efficient approach^5^. Primary healthcare (PHC) providers are ideally positioned to deliver BISC^6,7^ due to their credibility^8,9^ and frequent contact with patients, particularly those with conditions like diabetes and hypertension, who could greatly benefit from smoking cessation^10^ and those who frequent PHC clinics.

Despite BISC’s effectiveness and an appropriate health workforce, its implementation in China has been insufficient^11,12^. Clinical data show that the percentage of clinicians who actively ask whether patients smoked is low, and only about one-third (33.9%) of patients report receiving such advice^13^. This demonstrates that BISC has not yet become a routine practice.

This challenge highlights a crucial gap in China’s research landscape, which has largely focused on developing new interventions rather than effectively implementing existing ones. To address this, our type 3 hybrid effectiveness-implementation trial will apply a rigorous and innovative methodology.

This study use of the Multiphase Optimization Strategy (MOST) framework to optimize a tobacco cessation implementation strategy, providing a novel paradigm for future public health research. The methodology and findings from this study are expected to be highly relevant and adaptable to other low- and middle-income countries (LMICs) facing similar public health challenges. The primary objective of this study is to identify the optimal set of implementation techniques for promoting BISC based on prior research on barriers and facilitators.

## METHODS

### Study design

*Hybrid and full factorial design*

**Figure 1:**
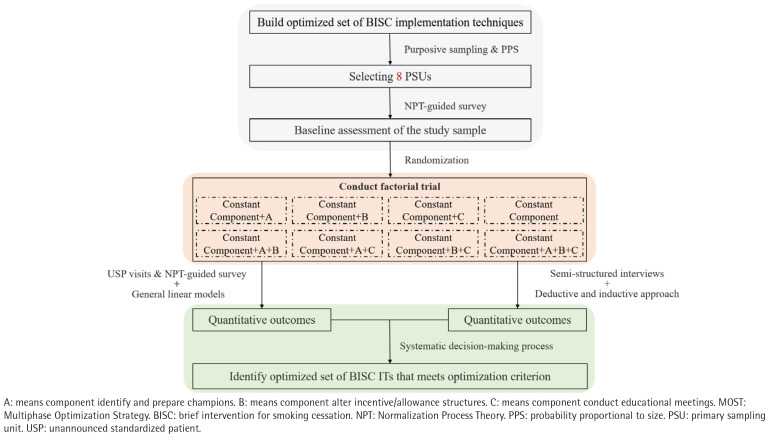
Study design of the MOST-based type 3 hybrid implementation trial using a factorial design (2×2×2) in primary healthcare institutions with a total sample of 640 clinicians, Guangdong Province, China, January 2026 – December 2026

This study is designed as a type 3 hybrid implementation trial^14^, concentrating on the development and assessment of the effectiveness of a specific implementation strategy to enhance the adoption and implementation of BISC. The design prioritizes the evaluation of implementation strategies while also examining clinical outcomes. The research adheres to the principles of the Multiphase Optimization Strategy (MOST)^15,16^ with its core component being a 2×2×2 full factorial design (Figure 1). This design will result in eight distinct experimental conditions and allows for the evaluation of the main effects and potential interaction effects of the implementation techniques, which is the core purpose of the optimization trial. The study has identified four key implementation techniques to be included in the trial. One of these, assessing readiness and identifying barriers and facilitators, will be included as a constant component in all eight conditions, ensuring a foundational level of support for all participants. The remaining three techniques serve as the three factors of the factorial design, each with two levels (present or absent) (Table 1).

**Table 1:** Full factorial design (2×2×2) with constant components within a type 3 hybrid effectiveness-implementation trial of BISC in primary healthcare facilities with a sample of 640 clinicians, Guangdong, China, January 2026 – December 2026

*Experiment* *conditions*	*Constant component*	*Optional components to be tested*		
	*Assess for readiness and* *identify barriers and* *facilitators*	*Identify and prepare* *champions*	*Alter incentive/allowance* *structures*	*Conduct educational* *meetings*
1	Yes			
2	Yes	Yes		
3	Yes	Yes	Yes	
4	Yes	Yes		Yes
5	Yes		Yes	
6	Yes		Yes	Yes
7	Yes			Yes
8	Yes	Yes	Yes	Yes

Yes: means included in intervention. No: means not included in intervention. The specifics form (such as ‘ranking primary healthcare clinicians’ execution of BISC and providing spiritual rewards for excellence’) of each component (such as ‘Alter incentive/allowance structures’) will be developed by inviting stakeholders to work together, following the principles of co-production. BISC: brief intervention for smoking cessation.

*Randomization*

Following multistage and clustered sampling, the allocation sequence will be generated using a random number table. Simple randomization for each group will be conducted independently by a statistician not affiliated with the research team. The randomization scheme will be sealed in an envelope and opened only at the time of formal randomization, enabling the researcher to execute the random assignment.

*Blinding*

Blinding will not be feasible for the researchers and study subjects (institutions and clinicians) as both groups will actively participate in implementing the assigned interventions based on their allocation status. To mitigate subjective analysis, outcome assessors and data analysts will be blinded. Upon completion of all unannounced standardized patient (USP) visits for clinicians and telephone callback for patients, the research team will disclose the participants’ allocated interventions. Institutions will be decoded before analysis, and the allocation status will not be revealed to the data analysts throughout the trial.

### Recruitment and informed consent

*Study setting*

The study will be conducted in primary healthcare institutions located in Guangdong Province, China, from January 2026 to December 2026. To ensure the generalizability of our findings across the diverse socioeconomic landscape of the country, we will strategically select institutions from three cities that represent a spectrum of economic development: a highly-developed city from the Pearl River Delta region, a moderately-developed city from either Eastern or Western Guangdong, and a less-developed city from Northern Guangdong. This deliberate selection allows us to rigorously test our implementation strategies under a wide range of conditions, from highly-resourced urban settings to more resource-constrained rural areas.

The selected institutions will include a variety of primary care settings, specifically community health service centers and township health centers/village health stations, to ensure a broad representation of primary care delivery models. We will recruit a sufficient number of institutions to meet the sample size requirements determined in the section sample size and power.

The institutional inclusion criteria are as follows: 1) the institution must have been a member of a compact medical consortium within the last three years; and 2) the institution must provide comprehensive primary healthcare services. The exclusion criteria for institutions include: 1) ongoing participation in any other smoking cessation intervention studies; and 2) ongoing participation in other implementation studies that are testing any of the specific implementation techniques used in our trial.

*Study population*

The study population is clinicians, encompassing licensed clinicians, licensed assistant clinicians, and certified village clinicians. Clinicians will be included based on the following criteria: 1) they have worked in a clinical capacity for at least six months; and 2) they provide outpatient and primary healthcare services. Conversely, clinicians will be excluded if they meet any of the following criteria: 1) they have previously received smoking cessation-related training or participated in a similar trial; 2) they are absent from work for one month or more; 3) they are a non-formal employee such as a temporary worker or rotational staff; and 4) they are currently participating in other studies related to any of the specific implementation techniques being tested in the present trial.

*Sampling procedure*

**Figure 2:**
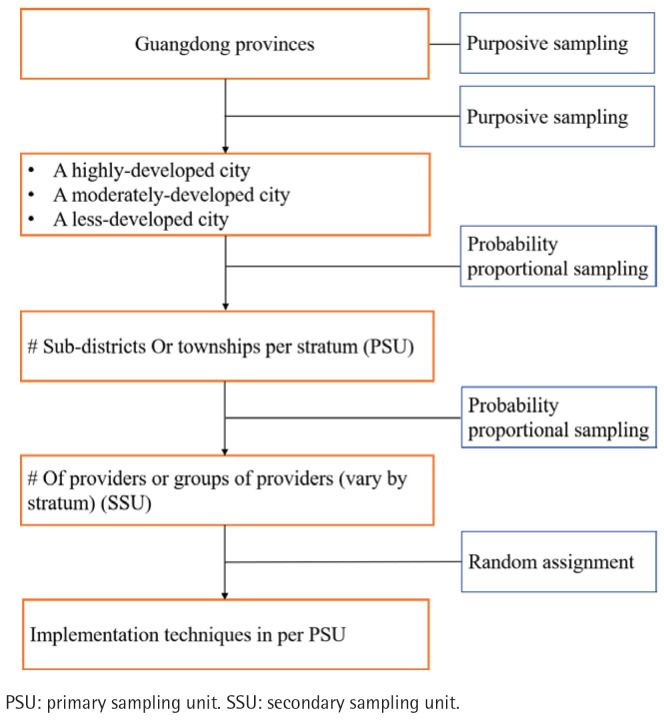
Sampling procedure of primary healthcare facilities with 640 clinicians, Guangdong Province, China, January 2026 – December 2026

This study will use a multistage, clustered sample design. The primary sampling unit (PSU), which is a regrouped unit of clinics or facilities, will serve as the randomization unit. The sampling process will be conducted in multiple stages (Figure 2). The first stage involves the purposive selection of three cities within Guangdong province to ensure a comprehensive representation of different socioeconomic development levels. Specifically, we will select one highly-developed city from the Pearl River Delta region, one moderately-developed city from either Eastern or Western Guangdong, and one less-developed city from Northern Guangdong.

In the second stage, all primary healthcare institutions under the jurisdiction of the recruited local health authorities in these three cities will be included in the sampling frame. These institutions will then be regrouped into PSUs based on the number of clinicians. This ensures that each unit has a sufficient number of clinicians, preventing the exclusion of smaller institutions from the sampling process. The PSUs will then be sampled using a probability proportional to size (PPS) sampling method.

The study will be conducted as a cluster-randomized trial. The intervention will be delivered to the entire cluster (PSU), but the intervention’s effect will be measured at the clinician level. The measurement is based on the average performance of a random sample of clinicians within the cluster. This is achieved through unannounced standardized patient (USP) visits, which function as the final step of the sampling process. A minimum of 80 USP visits will be assigned to each experimental condition.

*Sample size and power*

Power analyses were conducted to determine the sample size needed to test the effect of the implementation strategies on our primary outcome, ‘clinician advice to quit’. The detailed definition and measurement of this outcome are provided in the section on study outcomes. Based on prior research, the baseline rate for this outcome was 33.9%^13^. Through stakeholder consultation, it was determined that a 40% increase in delivery, resulting in a final primary outcome of 47.46%, would be considered a clinically meaningful effect. Assuming a two-tailed test with an alpha level of 0.05 and a desired power of 0.8, a sample size of 28 participants per cluster is required. Accounting for the cluster-randomized design with a design effect (Deff) of 2 and adjusting for an anticipated 20% loss to follow-up, the sample size has been intentionally increased to further enhance statistical power for detecting higher-order interactions. Consequently, the required sample size for one experimental condition is approximately 80 participants. Given our study design with eight conditions (one condition per PSU, for a total of eight PSUs), this translates to 640 participants in total.

### Core intervention

Brief verbal intervention for smoking cessation (BISC), which is a 1–3-minute verbal advice delivered by health professionals during routine consultations, is a highly effective and cost-efficient approach. Based on the China Clinical Smoking Cessation Guidelines, BISC includes: asking about smoking status, asking about the number of cigarettes smoked, discussing the harms and benefits of quitting, advising to quit, and providing a referral.

### Implementation strategies

*Optimization conceptual model*

**Figure 3:**
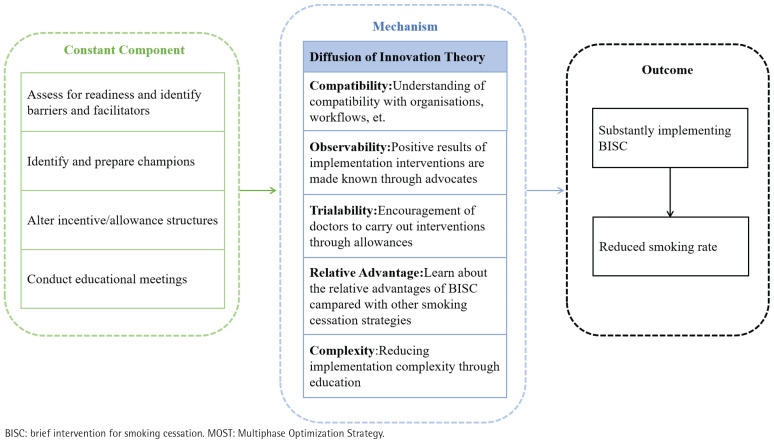
Conceptual model for optimizing BISC implementation strategies within the MOST framework in primary healthcare, Guangdong, China

An optimization conceptual model for the implementation of BISC was constructed, employing mixed methods guided by Diffusion of Innovation Theory. Based on this theory, attributes of an intervention strategy can facilitate intervention implementation, including compatibility, observability, trialability, relative advantage, and complexity. This conceptual model has integrated elements and their respective levels of implementation techniques and mechanisms that influence the implementation of BISC. We have developed a preliminary conceptual model based on our current understanding (Figure 3), which has undergone further refinement.

*Implementation strategy function*

The first phase of this study, which has been completed, was designed to systematically identify a set of implementation techniques for the optimization trial (Supplementary file Appendix A).

*Implementation strategy forms*

Following the identification of the strategy functions, the specific forms (i.e. the detailed actions and activities) for each strategy were developed and specified to ensure local relevance and feasibility. This process was guided by recommendations from the Proctor Implementation Strategy Reporting Specification^17^, and involved phased stakeholder interviews to validate and refine the checklist of strategies (Supplementary file Appendix B). The study subsequently involved stakeholders in identifying the specific forms (representing levels of implementation techniques), adhering to the principle of co-production. Discussions with stakeholders guided the selection of implementation techniques that were deemed consistent across all sites and those that were optional for testing in the factorial trial. The selection of constant components was based on ethical or logistical considerations, ensuring that specific intervention elements were uniformly delivered to all participants.

### Constant component

*Assessing readiness and identifying barriers and facilitators*

The strategy of assessing readiness and identifying barriers and facilitators will be included as a constant component across all eight experimental conditions. This ensures a baseline understanding of the implementation environment and addresses fundamental challenges uniformly across all participating sites.

A proactive assessment of the readiness of primary healthcare institutions and clinicians to implement BISC was conducted through an online questionnaire. This was completed within the first week of the project commencing. Conduct monthly online questionnaire and online focus group discussions to identifying contextual barriers and facilitators.

### Optional components

*Incentives*

This strategy aims to enhance BISC implementation through a multi-pronged incentive approach, primarily targeting clinicians and organizational commitment.

*Foundational training*

Within the first week of the project intervention commencing, third-party experts will conduct a one-time training for all medical staff, focusing on BISC skills. This pre-implementation incentive aims to enhance the hospital’s overall knowledge and skills regarding BISC.

*Issue an administrative directive*

Hospital administrators and middle-level leaders (e.g. department heads) will issue a unified administrative directive within 24 hours of foundational training, mandating clinicians to actively perform BISC. This action aims to ensure low time-cost for clinicians.

*Assessment and public disclosure*

The execution of BISC will be incorporated into medical service quality assessments. Weekly, in conjunction with medical quality surveys, the Medical Affairs Department or a third party will conduct random patient follow-up checks to assess BISC implementation, and public the results.

*Identifying and preparing champions*

This strategy focuses on establishing internal champions within the institutions to drive BISC implementation. The timeline for this strategy will be extended to ensure effective engagement and training.

*Identification*

Recruitment calls will be issued to all hospital staff, offering research funding as an incentive to attract promoters. This should be conducted within one week of the project commencing.

*Selection and empowerment*

Following identification , one-on-one motivational interviews will be conducted with interested candidates to help them understand the role. After selection, formal promoters will be appointed based on these interviews and administrative assessments. This combined approach provides a balanced method for selecting capable and motivated individuals.

*Cultivation*

This is initiated immediately after empowerment and continues throughout the entire project cycle. Identified promoters will receive BISC-related learning materials and begin participating in practical projects with the project team. This will involve monthly participation opportunities in communication and training processes, and the promoters will begin organizing small-scale internal trainings within their departments. This aims to deepen their understanding and improve overall implementation efficiency.

### Education and training

This strategy focuses on enhancing the knowledge and skills of all medical staff through structured educational activities. The frequency of these activities is designed to build momentum while avoiding an excessive burden on staff.

*Specialized workshops*

Starting from the first month, the project team will conduct periodic thematic workshops on BISC-related knowledge and lectures on execution for all hospital staff. These will be held monthly after the initial appointment of promoters. These will be held once a month.

*Embedded health education*

Starting from Week 1, it is imperative to reinforce the knowledge of tobacco control among healthcare professionals on a daily basis, utilizing a variety of concise formats to ensure effective dissemination of information.

*Training exchange*

Starting from Week 8, public health departments and department heads will organize periodic smallgroup salon activities and peer-sharing sessions for all hospital staff.

### Strategy coordination

The phased timeline for these strategies is designed to be realistic and manageable for clinicians in a busy primary care setting. The activities are staggered, with promoter identification taking place first, followed by a more gradual rollout of thematic training and exchange sessions to avoid overwhelming staff and to facilitate effective skill building over time.

### Ethical considerations

The study has been approved by the Biomedical Ethics Committee of Southern Medical University. A waiver of informed consent for obtaining service owner or clinician participation has also been granted. This waiver is crucial to mitigate potential observation bias, such as the Hawthorne effect^18^, as the use of USPs requires that clinicians are unaware they are being evaluated.

The institutional review board will oversee the study, ensuring that the rights of human subjects are protected. A Data and Safety Monitoring Board (DSMB) will be used to provide independent supervision. While minimal risks are associated with this study, any adverse events will be reported to the appropriate investigators and institutional review board as per standard procedures. Furthermore, all research staff will be trained on the importance of privacy. To protect participant confidentiality, a unique research ID will be assigned to each participant, and all data will be stored in password-protected electronic files or locked cabinets. Data extracted from medical records will not be linked to patient-identifying data. Moreover, all audio recordings will be erased at the end of the project.

### Study variables

*Demographic variables*

Demographic variables include institution information, clinician sociodemographics, and patient sociodemographics. Detailed variables, data collection methods, and time points are summarized in Table 2.

**Table 2:** Study variables of the type 3 hybrid effectiveness-implementation trial, Guangdong, China, January 2026 – December 2026

*Category*	*Outcome* *type*	*Variable*	*Theoretical basis*	*Definition*	*Variable type*	*Tool*	*Acquisition* *methods*	*Study periods ^a^*				
								*I*	*II*	*III*	*IV*	*V*
**Basic information**	Institution information	Institution location, level, etc.		-	-	-	Questionnaire survey	●				
	Socio-demographic of clinician	Gender, age, title, department, etc.		-	-	-	Questionnaire survey	●				
	Socio-demographic of patient	Gender, age, primary diagnosis, etc.					Telephone callback		●	●	●	●
**Implementation outcomes**	Primary outcome	Clinician asks smoking status	IOF: Fidelity RE-AIM: Implementation	From the first step in the BISC implementation steps in the China Clinical Cessation Guidelines (2015 edition), indicating whether the clinician asked smoking status or not.	Quantitative data, dichotomous variable (0,1)	Questionnaire	USP visit/telephone callback^b^		●	●	●	●
		Clinician advises to quit	IOF: Fidelity RE-AIM: Implementation	From the fourth step in the BISC implementation steps in the China Clinical Cessation Guidelines (2015 edition), indicating whether the clinician advised to quit.	Quantitative data, dichotomous variable (0,1)	Questionnaire	USP visit/telephone callback^b^		●	●	●	●
	Secondary outcome	Clinician asks about number of cigarettes smoked	IOF: Fidelity RE-AIM: Implementation	From the second step in the BISC implementation steps in the China Clinical Cessation Guidelines (2015 edition), indicating whether the clinician asked about number of cigarettes smoked.	Quantitative data, dichotomous variable (0,1)	Questionnaire	USP visit/telephone callback		●	●	●	●
		Clinician discusses the harms and benefits of quitting	IOF: Fidelity RE-AIM: Implementation	From the third step in the BISC implementation steps in the China Clinical Cessation Guidelines (2015 edition), indicating whether the clinician told about harms/benefits of quitting.	Quantitative data, dichotomous variable (0,1)	Questionnaire	USP visit/telephone callback^b^		●	●	●	●
		Clinician provides a referral	IOF: Fidelity RE-AIM: Implementation	From the fifth step in the BISC implementation steps in the China Clinical Cessation Guidelines (2015 edition), indicating whether the clinician referral patient to smoking cessation clinic.	Quantitative data, dichotomous variable (0,1)	Questionnaire	USP visit/telephone callback^b^		●	●	●	●
		Coverage of BISC delivery	IOF: Penetration RE-AIM: Reach	Percentage of patients who received BISC as compared to the patients who attended to PHC.	Quantitative data, percent (0–100%)	Questionnaire	USP visit/telephone callback^b^		●	●	●	●
	Tertiary outcome	Applicability of BISC delivery	IOF: Feasibility	The extent to BISC can be successfully carried out within PHC clinician.	Quantitative data 0–100 points	NPT questionnaire	Questionnaire survey				●	
		Acceptance of BISC delivery	IOF: Acceptability REAIM: Adoption, Maintenance	The perception among the PHC facilities that BISC implementation techniques is agreeable, palatable, or satisfactory.	Qualitative data	Interview outline	Semi-structured interview				●	
			IOF: Adoption RE-AIM: Adoption	The intention, initial decision, or action to try or employ BISC from the perspective of the PHC facilities.							●	
			IOF: Appropriateness RE-AIM: Adoption	The perceived fit, relevance, or compatibility of BISC for the PHC facilities; and/or perceived fit of BISC to address a particular issue or problem.							●	
		Sustainability of BISC delivery	IOF: Sustainability RE-AIM: Adoption, Maintenance	The possibility to BISC is maintained or institutionalized within PHC clinician’s ongoing, stable operations.	Quantitative data	The Program Sustainability Assessment Tool (PSAT)	Questionnaire survey				●	
		Sustainment of BISC delivery	IOF: Sustainability; RE-AIM: Maintenance	The continued implementation of BISC by the primary care clinician over time.	Quantitative data and qualitative data	The Provider Report of Sustainment Scale (PRESS) and interview outline	Questionnaire survey and semi-structured interview					●
		Cost of BISC delivery	IOF: Implementation Cost RE-AIM: Implementation	The cost of implementation, depends upon the costs of the BISC implementation techniques.	-	Administrative data					●	
**Effectiveness outcomes**	Secondary outcome	7-day point abstinence status		Self-reported abstinence from smoking for the past 7 days.	Quantitative data and qualitative data	-	Telephone callback			●	●	●
		Daily cigarette consumption		Number of cigarettes smoked daily. To assess a reduction in smoking behavior.	Quantitative data and qualitative data	-	Telephone callback			●	●	●
	Tertiary outcome	Patient satisfaction with the BISC provided by the clinician		The patient’s perceived satisfaction with how the physician implemented BISC during the consultation.	Quantitative data and qualitative data	-	Telephone callback			●	●	

a I: Enrolment. II: Baseline (i.e. before the first of BISC implementation). III: Mid-term (i.e. at the middle of BISC implementation), which will be executed the weeks 1, 3, 5, 7, 9 and 11 after the start of the BISC implementation. IV: Endline (i.e. at the end of BISC implementation). V: Follow-up (i.e. maintenance of the BISC), which will be executed 6 months after the endline.

b Data collection via telephone callbacks with patients in periods I and V; Data collection by USP visits in period IV. IOF: Implementation Outcomes Framework. BISC: brief intervention for smoking cessation. USP: unannounced standardized patient.

*Outcomes*

The study outcomes will be aligned with the principles of Proctor’s Implementation Outcomes Framework (IOF) and the RE-AIM framework. According to the type 3 hybrid design, the outcomes are categorized into primary and secondary aims to reflect the dual focus on implementation strategies and patient-level effects.

Primary aim: implementation outcomes

The primary aim of this study is to evaluate the effectiveness of the implementation strategies. These outcomes will be assessed by a combination of methods to ensure a comprehensive evaluation.

The primary outcomes, ‘clinician ask smoking status’ and ‘clinician advise to quit’, will be assessed using unannounced standardized patient (USP) visits. These outcomes serve as key measures of fidelity to the brief intervention protocol. The secondary outcomes, ‘clinician ask about number of cigarettes smoked’, ‘clinician discuss the harms and benefits of quitting’, and ‘clinician provide a referral’, will also be measured using a combination of USP visits for clinicians and telephone callbacks for outpatients.

The use of USPs is intended to mitigate observation bias and provide a valid, objective, and precise measurement^18,19^. We will employ three USP cases involving male current smokers with hypertension, type 2 diabetes, and cold, all requiring clinicians to provide BISC per national clinical practice guideline (Supplementary file Appendix C). Previously, our study developed, validated, and implemented 11 USP cases^20^ to evaluate the quality of PHC services, which contain a quality assessment checklist for the correct disposition of the physician in the desk design (Supplementary file Appendix D). These cases are reflective of the most prevalent conditions encountered in the PHC provider. The present study employed a standardized and well-established USP recruitment, training, and quality-control system based on the research team’s prior USP-based projects. Based on our previous experience, where a single USP can complete up to 72 visits per month, recruiting 20 USPs is sufficient to cover all 640 clinicians. In total, 20 USPs will be recruited across three regions, with approximately 6–7 USPs allocated to each city. All clinicians in the PSUs (a total of 640) will receive at least one USP visit. Each USP is scheduled to conduct a total of 32 visits. USP assignments to clinicians and clusters are performed using a random number table to ensure randomization and balanced coverage across clusters.

The sustainability of BISC delivery, defined as the likelihood of the intervention being maintained in practice, will be measured using the Program Sustainability Assessment Tool (PSAT)^21^. The sustainment of BISC delivery, or continued implementation over time, will be measured six months after the intervention. As USP visits are not suitable for long-term follow-up due to the risk of the standardized patients being recognized by clinicians, we will measure sustainment using the Provider Report of Sustainment Scale (PRESS)^22^ and semi-structured interviews to assess clinicians’ self-reported behavior and perceptions. The appropriateness of BISC delivery will be measured by Normalization Process Theory (NPT)-guided questionnaires (Supplementary file Appendix E)^23^, while its acceptability will be assessed through semi-structured interviews. Lastly, costs associated with the implementation strategies will be measured using administrative data.

Secondary aim: effectiveness outcomes

The secondary aim of this study is to assess the effects of the BISC intervention on patient-level outcomes. These outcomes will be measured via a patient phone call survey at two distinct time points. At the end of the intervention, a phone call will be made to all patients who were identified as smokers and received the BISC intervention during the study period. This initial survey will assess their smoking status immediately following the intervention and confirm their receipt of the BISC components. A 6-month follow-up survey will then be conducted exclusively with patients who self-reported as smokers and received the BISC intervention. This survey will assess the long-term effectiveness of the intervention by measuring key indicators, including 7-day point abstinence status, daily cigarette consumption, and patient satisfaction with the BISC provided by the clinician. A summary of all outcome measures, their respective assessment methods, and time points is presented in Table 2.

### Data analysis

*Quantitative analysis*

Data will undergo de-identification prior to analysis. Baseline characteristics of participants will be presented, utilizing mean and standard deviation for continuous variables and frequency and percentage for categorical variables. The trial outcome analysis will adhere to the intent-to-treat principle. Generalized estimating equations (GEE) with a binomial distribution and log link function will be used for the primary binary outcome, with clustering specified at the PSU level, to assess the main and interaction effects of the four implementation techniques on adherence to BISC practices among primary healthcare clinicians. The model will account for robust predictors of outcomes, including baseline adherence scores and sociodemographic factors of clinicians^24-26^. Effect coding will be applied to each experimental condition, and subgroup analyses based on age, gender, clinician level, and facility will be conducted to further explore the impact of different technique combinations on BISC implementation. Sensitivity analyses, involving imputation of missing values through random forest, will be performed to validate the robustness of the findings^27^. For continuous outcomes, GEE models with a Gaussian distribution and identity link function will be employed, adjusting for the same covariates and clustering at the PSU level. In the analysis, control for covariates including clinician-related information (gender, age, professional title, and department) and patient-related information (gender, age, primary diagnosis, and smoking status). We will perform subgroup analyses on patient smoking status, level of tobacco dependence, and clinician department to investigate differences in BISC implementation among different types of clinicians for various types of patients.

Hypothesis tests will be two-sided, with statistical significance set at p<0.05. All statistical analyses will be performed using Stata version 16.0 and R version 4.5.2.

*Qualitative analysis*

Qualitative data analysis from stakeholders will be de-identified and imported into Nvivo 11.0 for transcription, organization, coding, and content analysis. Our qualitative data analysis will use a mixed approach, combining both deductive and inductive methods, to identify established themes, patterns, and new discoveries^28,29^. For the first phase of the study, data from our scoping review^30^ and stakeholder consultations were analyzed deductively using the Consolidated Framework for Implementation Research (CFIR)^31^ to identify barriers and facilitators. For the second phase, data from semi-structured interviews will be analyzed inductively to find new themes. These data will also be analyzed using NPT to evaluate how the BISC intervention is integrated and sustained in practice. To ensure our coding is reliable, two team members will independently code each interview, and any differences will be resolved through discussion.

The systematic decision-making process^32^, guided by data from the factorial trial, will be integral to this procedure. We will leverage the estimated main effects to identify techniques that have a statistically significant positive impact. We will also examine interaction effects to glean insights into potential synergies or antagonisms between the techniques. The final optimization of implementation strategies will be achieved by integrating information from both main and interaction effects, along with a consideration of the predetermined constraints of the optimization objective. This approach ensures that the final selection of techniques is the most effective and efficient combination.

## DISCUSSION

### Overview and study purpose

Brief verbal intervention for smoking cessation (BISC) is considered an evidence-based practice (EBP) that combines effectiveness and affordability, and primary care physicians were considered suitable for participation in the intervention^6,8,9^. However, BISC is currently being implemented inadequately by primary care physicians in China. Developing, evaluating and adjusting multifaceted implementation strategies is crucial for translating EBP into clinical practice. This proposed study seeks to identify the most effective combination of implementation techniques to enhance adherence to BISC among primary care clinicians in China, while considering contextual constraints. The study is designed as a type 3 hybrid trial, which allows us to concentrate on implementation strategies for a proven best practice, thereby filling a notable gap in implementation research in China. This protocol provides a rigorous and innovative blueprint for advancing implementation science research in primary healthcare.

### Strengths and limitations

The study’s methodology possesses several significant features that advance implementation science. Traditional approaches often involve the simultaneous use of multiple, fragmented implementation strategies as part of an intervention, making it difficult to determine the impact of individual strategies or how they interact. The MOST framework overcomes these limitations by optimizing implementation strategies. Using a factorial design, it can identify the specific components of an intervention package that are effective, while balancing effectiveness against affordability, scalability, and efficiency. This pragmatic approach ensures the resulting strategies are both feasible and sustainable by proactively optimizing them within constraints, such as resource limitations. Moreover, the study’s dual theoretical framework, utilizing CFIR for determinant analysis and NPT for process and outcome analysis, provides a systematic and comprehensive approach to evaluating key implementation factors^33,34^. The use of USPs as a core measurement tool is another significant methodological contribution. By mitigating observation bias, USPs provide a more valid and objective assessment of BISC implementation fidelity than self-report or observed measures. This robust measurement strategy is crucial for accurately evaluating the effectiveness of the implementation strategies and ensuring the reliability of the study’s findings. This study has the potential to provide a valuable methodological reference for LMICs facing similar tobacco control situations.

The study acknowledges its limitations. Our focus on a factorial design means we are evaluating the average treatment effect of implementation strategies. While this approach aims to derive an optimal combination of techniques, it is important to acknowledge that implementation is frequently influenced by specific contextual factors that may not be fully captured. The implementation strategy’s effectiveness is therefore not necessarily applicable to other settings. A further limitation is our reliance on unannounced standardized patient (USP) visits for objective measurement of clinician behavior at a single time point. As USPs are not suitable for long-term follow-up due to the risk of the standardized patients being recognized, we are unable to objectively measure the sustainment of clinician behavior. We address this by using self-reported scales and interviews, but a degree of reporting bias may be present.

## CONCLUSIONS

To navigate the balance between creating generalizable knowledge and meeting context-specific implementation needs in implementation science, we will conduct a series of studies. In our forthcoming trial, we will delineate the functions of the intervention, which will undergo testing, while the forms (which represent the levels in the current study) will be tailored to suit the specific context^35^. This approach, which we term ‘precision implementation’, may serve as a methodological paradigm for other low- and middle-income countries. Ultimately, this could help address the significant public health threat of tobacco consumption in China and other LMICs^36^.

## Supplementary Material



## Data Availability

Datasets used and/or analyzed in the current study will be made available by the corresponding author upon reasonable request. All data generated or analyzed during this study are with the corresponding author. He is available to answer any questions about the datasets. Persons who have made outstanding contributions or assisted in this study may apply for the use of the data only after submitting the study hypothesis and signing a data confidentiality agreement. There is no fee for the data opening plan. Publication of the study results will include processed data only, and personal information will remain confidential.
