# Supplementary material for: Multiphase optimization implementation strategy for tobacco cessation interventions in primary care clinics: A cluster-randomized type 3 hybrid effectiveness-implementation trial
Source: Tob Induc Dis. 2026 Mar 24;24:10.18332/tid/217143. doi: 10.18332/tid/217143 (PMC13012015; doi:10.18332/tid/217143)
Supplement: Supplementary file 1 [file TID-24-43-s1.pdf]

Appendix

Appendix A Implementation Strategy Function

Appendix B Core implementation strategies for brief smoking cessation intervention programmes based on the Proctor report specifications

Appendix C Clinical scenario: a typical middle-aged undiagnosed type 2 diabetes Unannounced Standardized Patient (USP)

Appendix D Quality assessment checklist: a typical middle-aged undiagnosed type 2 diabetes USP

Appendix E Questionnaire on the degree of implementation of regularization of BISC based on NPT

Appendix A Implementation Strategy Function

This process, guided by a scoping review(1), stakeholder consultations, and a Best-Worst Scaling (BWS) online survey(2), identified four key strategy functions to be included in the factorial trial: altering incentive/allowance structures, identifying and preparing champions, conducting educational meetings, and assessing readiness and identifying barriers and facilitators.. Based on ethical and logistical considerations, the " assessing readiness and identifying barriers and facilitators " strategy will be a constant component, while the other three will serve as the optional components to be tested for their main and interaction effects.

The optimization objectives for this study were definitively established, based on the specific implementation outcomes and constraints. The objective was to identify the most effective implementation strategy by balancing optimal outcomes with the established limitations, rather than solely maximizing outcomes. Through a stakeholder focus group, the barriers to BISC implementation were identified. These non-modifiable or difficult-to-modify barriers were then directly translated into the optimization constraints for the trial, with a specific focus on a cost constraint of 100 RMB and a time constraint of 3 minutes.

Reference

1. Siyuan L, Chen J. Using Consolidated Framework for Implementation Research to investigate facilitators and barriers of implementing Brief Intervention for Smoking Cessation among healthcare providers: a scoping review2025.
2. Chen J, Wang L. Stakeholder-Driven Priorities for Implementing Brief Smoking Cessation Interventions: A Best-Worst Scaling Approach2025.

Appendix B Core implementation strategies for brief smoking cessation intervention programmes based on the Proctor report specifications

Strategy	Action	Actor	Action Target	Temporality	Dose	Affected implementation outcomes	Reason
**A.Assessing Readiness and Identifying Barriers and Facilitators**	**A1. Assessing readiness:** Employ the validated, Chinese-translated Organisation Readiness for Evidence-Based Practice (ORIEBP) assessment tool (5 dimensions, 32 items, 5-point Likert scale plus 0/1 binary scoring). Collect data from all clinicians and managers within each organisation to calculate the institutional-level score (total item score divided by 32).	Project team assessors and institutional information officers working in pairs.	All medical staff in clinical departments, including doctors and nurses.	Completed within the first week of project commencement; Online questionnaire: 3 days; Paper-based follow-up: 2 days.	One-off comprehensive assessment for all staff; each institution must achieve a response rate of ≥80%.	The likelihood of brief smoking cessation interventions being adopted by organisations, implemented faithfully, and sustained over time to enhance institutional uptake, standardised implementation, and long-term execution.	Standardised measurement tools enable the swift acquisition of comparable data, allowing management to rapidly identify shortcomings and make resource allocation decisions.
	**A2. Identification of Barriers and Facilitating Factors:** Establish a 'Barriers-Facilitators Rapid Reporting Form' mini-programme, enabling doctors and nurses to instantly tick boxes or upload text detailing implementation barriers (e.g., time conflicts, patient resistance) and facilitating conditions (e.g., departmental support, bonus incentives). Conduct monthly 30-minute online focus group discussions (6–8 participants per group) for in-depth interviews.	Medical Department or third-party assessment agency.	All medical staff in clinical departments, including doctors and nurses.	The intervention is ongoing throughout the entire process; data from the previous month is collected on the first working day of each month, with interviews completed during the second week.	One online questionnaire per month (2 minutes) + one focus group session per month (30 minutes).	Ensure real-time correction and optimisation of the intervention process to enhance long-term adherence.	Dynamic identification of obstacles and timely iteration of strategies can significantly enhance the sustainability of interventions.
**B.Incentives**	**B1. Foundational Training:** Training content includes the evidence-based medical rationale for brief smoking cessation interventions (e.g., the extent to which they reduce risk), case studies of successful outcomes (e.g., a clinician assisting a patient through brief cessation support), tobacco	Third-party experts or project teams.	All medical staff in clinical departments, including doctors and nurses.	Within the first week of the project intervention commencing, prior to the issuance of administrative documents by B2.	A one-off training session lasting 45 minutes, followed by a 15-minute Q&A session, with a total duration not exceeding one hour.	Enhance healthcare professionals' readiness for compliance and acceptance of interventions.	Communicating the evidence-based value of interventions to healthcare professionals to alter their negative perceptions regarding low cost-effectiveness. This constitutes a low-cost, high-efficiency initial intervention capable of rapidly reaching a large number of medical staff.

	control policies and clinical guidelines, and concise operational procedures (e.g., the 2A+R and 4A+R models).						
	**B2. Issue an Administrative Directive:** Issue a notice entitled‘Promoting Brief Smoking Cessation Interventions by Clinicians During Outpatient Consultations’ in the form of an administrative directive. This notice shall clarify that brief smoking cessation interventions constitute an integral part of routine clinical practice and specify that compliance will be subject to random inspections and public disclosure.	Hospital administrators, middle management, department heads, etc.	All clinicians.	Issued within 24 hours of B1 training.	A formal, clear administrative document accompanied by operational guidelines.	Ensure the initiation rate and initial adherence to brief smoking cessation interventions.	By leveraging the coercive force of institutional mechanisms, interventions are transformed from optional measures into mandatory requirements, thereby fundamentally eliminating implementation barriers stemming from physicians' lack of external motivation. This approach incurs no additional costs, relying solely on hospitals' existing administrative management systems, rendering it highly feasible.
	**B3. Assessment and Public Disclosure:** Incorporated into existing grassroots healthcare service quality assessment programmes, this involves conducting regular telephone follow-ups with patients to gauge satisfaction levels and monitor outpatient clinicians' implementation of brief smoking cessation interventions. Performance metrics for randomly selected departments and clinicians shall be published periodically.	Medical Department or third-party assessment agency.	Departments and individual clinicians.	Following the issuance of B2 administrative documents, random inspections shall be conducted every Sunday, with results published on Mondays, concurrently with medical quality investigations.	Each week, the departments with medical records failing random inspections shall be publicly announced, and the department heads shall be provided with specific lists of non-compliant doctors. Each month, no more than three doctors demonstrating outstanding performance shall be selected for the title of ‘Smoking Control Pioneer’.	Ensure the continuity and integration of interventions.	By linking interventions to performance outcomes, this approach leverages external reinforcement and peer influence to motivate clinicians to sustain high-quality practice over the long term. Primarily utilising existing assessment frameworks, the measure incurs minimal costs while exerting a sustained positive influence on clinical behaviour, rendering it exceptionally cost-effective.
**C.Identifying and Cultivating Champions**	**C1. Identification:** Employing purposive sampling, formal recruitment notices for “Champions” shall be issued through internal management channels (such as the Medical Affairs Office and department heads), clearly outlining role responsibilities and	Managers or middle-level leaders within healthcare institutions (such as directors of medical affairs departments or heads of departments).	All clinicians within the healthcare institution who possess the potential willingness and capability.	To be conducted within one week prior to the project commencement.	One champion per sample hospital, and one champion per department or primary healthcare facility.	Enhance the endogenous capacity for interventions within the organisation.	Through proactive recruitment, healthcare professionals demonstrating enthusiasm and potential for tobacco control work are systematically identified, laying the groundwork for subsequent training initiatives. This represents a highly cost-

professional development benefits.						effective approach to talent discovery, requiring only the utilisation of existing internal communication channels.
**C2. Selection:** Conduct one-to-one motivational interviews with all candidates to explore their interests in depth and clarify the core responsibilities of the champion role.	Third-party experts or project teams, responsible for conducting professional interviews and assessments.	All candidates selected through the C1 stage.	Immediately following Phase C1, within the first week of the project.	Conduct one 20-minute one-to-one interview with each candidate.	Ensure high acceptance of the champion role and high adherence to intervention implementation.	Through one-to-one interviews, candidates' motivations and understanding are thoroughly assessed to ensure the final selection demonstrates a high degree of alignment. This approach offers cost control while significantly enhancing the success rate of interventions, thereby preventing resource wastage on unsuitable candidates.。
**C3. Empowerment:** Conferring authority and a sense of purpose upon champions through formal, public appointments.	Senior managers in healthcare institutions.	All selected champions.	Immediately following the C2 phase, within the first week of the project.	One formal letter of appointment, clearly outlining the core responsibilities of the promoter.	Enhance champions' sense of identity and acceptance of their role.	Through formal appointment, champions are provided with a clear identity and role, enabling them to work more effectively and gain recognition from colleagues. This initiative incurs no additional costs yet significantly boosts champions' intrinsic motivation and sense of responsibility.
**C4. Cultivation:** Deliver one core competency training session focusing on how champions can effectively provide peer support and overcome common obstacles encountered in grassroots practice. Provide ongoing online guidance centred on core responsibilities including demonstration, dissemination, support, coordination, advocacy and problem-solving, to continuously enhance champions' tobacco control knowledge and practical capabilities.	Third-party experts and project teams.	All selected champions.	Initiated immediately following Phase C3 and running throughout the entire project cycle. Core competency training commences in Week 1 of the project. During Weeks 2 to 12, champions enter the independent practice phase, with the project team providing weekly online	A intensive training session lasting 1–1.5 hours serves as the sole formal training for champions prior to commencing duties. Online guidance sessions, each lasting 10–15 minutes, are conducted to address critical issues encountered by champions during practical implementation.	Enhance champions' sense of self-efficacy and capacity for intervention implementation.	Through refined training and ongoing guidance, address technical challenges that champions may encounter while fulfilling their duties, ensuring they can effectively serve as role models and provide support. This model offers cost-effective solutions, particularly with online mentoring, which effectively supports promoters' continuous development and is well-suited to primary healthcare settings.

				guidance.			
**D.Education and Training**	**D1. Specialised Workshops:** Conduct small-scale, highly interactive workshops. These sessions focus on specific challenges within brief smoking cessation interventions, such as: 'How to skillfully initiate and broach the topic of tobacco control,''How to employ motivational interviewing techniques when encountering patient resistance,' 'How to assist patients in managing urges to relapse.'	Project team members, or specially trained and experienced in-house smoking control specialists.	All healthcare professionals requiring in-depth training in tobacco control intervention techniques, particularly key practitioners from various departments.	From the first month of the project, this will be repeated periodically, occurring once per month.	Each session lasts 1.5 hours, comprising 30 minutes of theoretical instruction followed by 60 minutes of practical exercises (such as scenario simulations and role-playing).	Addressing specific technical challenges encountered by doctors during interventions to enhance their sense of self-efficacy.	Through small-scale, highly interactive practical exercises, this approach helps clinicians master communication techniques in complex scenarios, thereby addressing shortcomings in foundational training. Whilst requiring a certain level of organisational effort, it efficiently resolves specific operational challenges faced by medical staff, offering a favourable return on investment.
	**D2. Embedded Health Education:** Continuously reinforce tobacco control knowledge within healthcare professionals' daily workflows through diverse bite-sized formats (e.g., electronic bulletins in work groups, morning meeting discussions, physical materials). Examples include: - Daily Tobacco Control Tips - Standard Responses to Patient Enquiries - Tobacco Control Conversation Cards - One-Minute Tobacco Control Flowchart	Public Health Department, project team, and heads of all departments.	All medical staff at the hospital.	Commencing from Week 1 of the project, this will be ongoing.	Fragmented channels shall convey a total of no fewer than 12 concise knowledge points.	Continuously instil and reinforce tobacco control concepts and techniques within the subconscious of healthcare professionals, transforming them into habitual thinking patterns thereby reducing the psychological burden in implementation.	Through fragmented, high-frequency communication, knowledge is integrated into daily workflows, ensuring healthcare professionals maintain continuous exposure to tobacco control information. This approach primarily leverages existing communication channels, incurring minimal costs while effectively reinforcing staff knowledge, rendering it highly feasible.
	**D3. Training Exchange:** An informal case review session conducted as an open discussion forum. Physicians voluntarily share real-world challenges encountered in practice, with colleagues collectively deliberating and identifying solutions.	Department heads or trained key personnel, who serve as the organisers and facilitators of the activities.	Small groups of medical staff, such as those within the same department or voluntary interest groups.	Concentrated implementation during the mid-project phase, specifically in Week 8.	Conducted as a single session, lasting 1-2 hours.	Establish a mutual learning mechanism to provide doctors with channels for resolving individualised issues.	Implemented midway through the project, this initiative aims to promptly address common issues encountered by healthcare personnel during initial practice. Through informal, peer-to-peer collaborative learning, it effectively supplements formal training, thereby enhancing the internalisation and effectiveness of interventions.

CLINICAL SCENARIO

SOCIAL-DEMOGRAPHIC PROFILE

Demographic profile: female/male around 55 years old, Han Chinese, university educated, married, with a daughter/child who is studying at university.
Social/economic: has medical insurance; family is relatively well-off with an annual income of about 180,000; has a car; has a three-bedroom house; usually lives in Changsha, Hunan.
Lifestyle: overweight: doesn't like to exercise; likes to eat meat, fewer vegetables and fruits; occasionally party and drink beer (about one bottle in a glass bottle): smokes half a pack of cigarettes every day for more than 20 years (men only); in his spare time, likes to play mahjong with friends.
Job: office clerk in a bank, about to retire; average work pressure.
Personality traits: not very talkative, but well-spoken.
Medical knowledge: largely lacking in medical knowledge.

CONTEXT

A few days ago he and his son/daughter went to visit his uncle's house/uncle's house as they happened to be passing by and, urged and accompanied by his daughter/son, went to the general medicine/internal medicine department (or village health office) of the primary health care facility where his uncle's house/uncle's precinct is located to seek medical treatment.
The patient is accompanied by one of the daughters/ sons. Both were dressed casually; the patient was in fair spirits, well-dressed, and polite in speech. Son/daughter is quiet and student-like, when asked about their relationship, son/daughter answers, when asked about the condition, the SP answers. (Passive lines)

COMPLAINT

One month ago presented with polyhydramnios, polyuria, and polyphagia for 1 month, with no relief, the condition worsened for 1 week.

EXAMINATION

Current blood pressure is essentially normal. The highest measurement before blood pressure management was 180/90 mmHg; no other abnormal signs.

LABORATORY & IMAGING

Carry the results of the medical examination for 1 month with you (take a picture of the paper version and store it on your mobile phone); Fasting venous plasma blood glucose 8.5mmol/L; Glycosylated hemoglobin 7.3%. Please refer to the medical examination report for details.

DIAGNOSIS

Type 2 diabetes; Grade 3 primary hypertension in the very high-risk group; Hyperlipidemia; Moderate to severe nicotine dependence (men)

Appendix C Clinical scenario: a typical middle-aged undiagnosed type 2 diabetes Unannounced Standardized Patient (USP)

Appendix D Quality assessment checklist: a typical middle-aged undiagnosed type 2 diabetes USP

**Item**		**Corresponding questions from doctors (Scoring points)**
**Quality of consultation and examination**		
Complaint &Current medical history	Drinking water (frequency, amount, etc.)	Approximately how much water can you drink per day? How much water did you drink in the previous day?
	Urination (frequency, volume, etc.)	Is the amount of urine about the same as the amount of water you drink or more? Number of the night starts?
	Remaining entries omitted	
Relevant medical history	Past medical history	What previous illnesses have you had?
	**Smoking status (if, how much, when, etc.)**	**Do you smoke? How many cigarettes per day? How many years have you smoked?**
	Remaining entries omitted	
Physical examination	Blood pressure	Need to record whether this test was performed and whether the test was performed in a standard way
	Skin elasticity (to determine the presence of dehydration)	Need to record whether this test was performed and whether the test was performed in a standard way
	Remaining entries omitted	
Laboratory tests & imaging	Fasting blood glucose	Points will be deducted for tests that are not required or are harmful if prescribed by the doctor
	Oral glucose tolerance test (OGTT)	Points will be deducted for tests that are not required or are harmful if prescribed by the doctor
	Remaining entries omitted	
**Diagnostic quality**		
Correct diagnosis	Type 2 diabetes	Extra Credit
	Remaining entries omitted	
Partially correct diagnosis	Diabetes or suspected diabetes	Extra Credit
	Remaining entries omitted	
Incorrect diagnosis	not acclimatized	Deductions
	Urinary tract infections	Deductions
	Remaining entries omitted	
**Quality of treatment**		
Correct treatment measures	Low-salt, low-sodium diabetic diet	Extra credit, details omitted
	**Telling about hazards of smoking, asking to quit smoking, making referrals and offering a quit line.**	**Points deducted if not implemented for male USP**, details omitted
	Remaining entries omitted	
	Insulin	Deductions
	Antibiotics	Deductions

Wrong treatment measures	Remaining entries omitted

USP, Unannounced standardized patient.

Appendix E Questionnaire on the degree of implementation of regularization of BISC based on NPT

**Construct**	**Sub-Construct**	**Item**
Coherence	Differentiation	1. I can see how the brief smoking cessation intervention differs from usual ways of working
	Communal specification	2. Staff in this organization have a shared understanding of the purpose of this brief smoking cessation intervention
	Individual specification	3. I understand how the brief smoking cessation intervention affects the nature of my own work
	Internalization	4. I can see the potential value of the brief smoking cessation intervention for my work
Cognitive Participation	Initiation	5. There are key people who drive the brief smoking cessation intervention forward and get others involved
	Enrolment	6. I'm open to working with colleagues in new ways to use the brief smoking cessation intervention
	Legitimation	7. I believe that participating in the brief smoking cessation intervention is a legitimate part of my role
	Activation	8. I will continue to support the brief smoking cessation intervention
Collective Action	Interactional workability	9.I can easily integrate the brief smoking cessation intervention into my existing work
	Relational integration	10. The brief smoking cessation intervention disrupts working relationships (reverse scoring)
		11. I have confidence in other people's ability to use the brief smoking cessation intervention
	Skill set workability	12 Work is assigned to those with skills appropriate to the brief smoking cessation intervention
		13. Sufficient training is provided to enable staff to use the brief smoking cessation intervention
	Contextual integration	14. Sufficient resources are available to support the brief smoking cessation intervention
		15. Management adequately support the brief smoking cessation intervention
Reflexive Monitoring	Systemization	16. I am aware of reports about the effects of the brief smoking cessation intervention
	Communal appraisal	17. The staff agree that the brief smoking cessation intervention is worthwhile
	Individual appraisal	18. I value the effects the brief smoking cessation intervention has had on my work
	Reconfiguration	19. Feedback about the brief smoking cessation intervention can be used to improve it in the future
		20. I can modify how I work with the brief smoking cessation intervention

BISC, Brief smoking cessation interventions; NPT, Normalization Process Theory.

CONSORT 2010 checklist of information to include when reporting a randomised trial*

Section/Topic	Item No	Checklist item	Reported on page No
**Title and abstract**			
	1a	Identification as a randomised trial in the title	Page 1
	1b	Structured summary of trial design, methods, results, and conclusions (for specific guidance see CONSORT for abstracts)	Page 2-page 4
**Introduction**			
Background and objectives	2a	Scientific background and explanation of rationale	Page 6
	2b	Specific objectives or hypotheses	Page 6-Page 7
**Methods**			
Trial design	3a	Description of trial design (such as parallel, factorial) including allocation ratio	Page 8-Page 9
	3b	Important changes to methods after trial commencement (such as eligibility criteria), with reasons	/
Participants	4a	Eligibility criteria for participants	Page 11
	4b	Settings and locations where the data were collected	Page 10-Pgae 11
Interventions	5	The interventions for each group with sufficient details to allow replication, including how and when they were actually administered	Page 14-Page 20
Outcomes	6a	Completely defined pre-specified primary and secondary outcome measures, including how and when they were assessed	Page 21-Page 24
	6b	Any changes to trial outcomes after the trial commenced, with reasons	/
Sample size	7a	How sample size was determined	Page 13
	7b	When applicable, explanation of any interim analyses and stopping guidelines	/
Randomisation:			
Sequence generation	8a	Method used to generate the random allocation sequence	Page 9
	8b	Type of randomisation; details of any restriction (such as blocking and block size)	Page 9
Allocation Concealment mechanism	9	Mechanism used to implement the random allocation sequence (such as sequentially numbered containers), describing any steps taken to conceal the sequence until interventions were assignedPage	Page 9

Implementation	10	Who generated the random allocation sequence, who enrolled participants, and who assigned participants to interventions	Page 9
Blinding	11a	If done, who was blinded after assignment to interventions (for example, participants, care providers, those assessing outcomes) and how	Page 9
	11b	If relevant, description of the similarity of interventions	/
Statistical methods	12a	Statistical methods used to compare groups for primary and secondary outcomes	Page 21-Page 22
	12b	Methods for additional analyses, such as subgroup analyses and adjusted analyses	/
**Results**			
Participant flow (a diagram is strongly recommended)	13a	For each group, the numbers of participants who were randomly assigned, received intended treatment, and were analysed for the primary outcome	/
	13b	For each group, losses and exclusions after randomisation, together with reasons	/
Recruitment	14a	Dates defining the periods of recruitment and follow-up	/
	14b	Why the trial ended or was stopped	/
Baseline data	15	A table showing baseline demographic and clinical characteristics for each group	/
Numbers analysed	16	For each group, number of participants (denominator) included in each analysis and whether the analysis was by original assigned groups	/
Outcomes and estimation	17a	For each primary and secondary outcome, results for each group, and the estimated effect size and its precision (such as 95% confidence interval)	/
	17b	For binary outcomes, presentation of both absolute and relative effect sizes is recommended	/
Ancillary analyses	18	Results of any other analyses performed, including subgroup analyses and adjusted analyses, distinguishing pre-specified from exploratory	/
Harms	19	All important harms or unintended effects in each group (for specific guidance see CONSORT for harms)	/
**Discussion**			
Limitations	20	Trial limitations, addressing sources of potential bias, imprecision, and, if relevant, multiplicity of analyses	Page 29-Page 30
Generalisability	21	Generalisability (external validity, applicability) of the trial findings	/
Interpretation	22	Interpretation consistent with results, balancing benefits and harms, and considering other relevant evidence	/
**Other information**			
Registration	23	Registration number and name of trial registry	Page 1-Page 42
Protocol	24	Where the full trial protocol can be accessed, if available	Pgae 2-

			Page 25
Funding	25	Sources of funding and other support (such as supply of drugs), role of funders	Page 32

*We strongly recommend reading this statement in conjunction with the CONSORT 2010 Explanation and Elaboration for important clarifications on all the items. If relevant, we also recommend reading CONSORT extensions for cluster randomised trials, non-inferiority and equivalence trials, non-pharmacological treatments, herbal interventions, and pragmatic trials. Additional extensions are forthcoming: for those and for up to date references relevant to this checklist, see www.consort-statement.org.

© 2026 Chen J. et al.
